# School Bullying, Self-Esteem, and Emotional Intelligence Among Primary Education Students: A Gender-Based Pretest Analysis from a School Nursing Perspective

**DOI:** 10.3390/healthcare14162571

**Published:** 2026-08-17

**Authors:** Malena Barba-Muñoz, María Pilar Molés-Julio, José Antonio Zafra-Agea

**Affiliations:** 1 Nursing Sciences, University Jaime I of Castellón, 12071 Castelló de la Plana, Spain; 2 Nursing Department, Faculty of Science Health, University Jaime I of Castellón, 12071 Castelló de la Plana, Spain; mjulio@uji.es; 3 Nursing Department, Faculty of Medicine, Autonomous University of Barcelona, 08193 Bellaterra, Spain

**Keywords:** school bullying, self-esteem, emotional intelligence, school nursing, primary education, mental health, bullying prevention, emotional health, gender differences

## Abstract

**Background:** School bullying represents a major public health challenge associated with adverse emotional and psychosocial outcomes during childhood. Self-esteem and perceived emotional intelligence may be relevant factors associated with bullying involvement. This study aimed to examine the relationships among school bullying, self-esteem, and perceived emotional intelligence in Primary Education students and to explore the indirect statistical associations between emotional intelligence dimensions and bullying involvement through self-esteem. **Methods**: A multicenter cross-sectional pretest study was conducted among 507 students from five educational centers in Madrid, Spain. School bullying was assessed using the Peer Harassment Questionnaire (PHQ), self-esteem using the Self-Esteem Questionnaire for Primary Education (A-EP), and perceived emotional intelligence using the Trait Meta-Mood Scale-24 (TMMS-24). Descriptive analyses, group comparisons, Pearson correlations, multiple linear regression, and exploratory mediation analyses were performed. Regression and mediation analyses were based on complete matched records. **Results:** Moderate bullying involvement was the predominant profile (51.3%), while 12.8% of students were classified as having high bullying involvement. School bullying was negatively correlated with self-esteem (*r* = −0.42, *p* < 0.001), emotional attention (*r* = −0.18, *p* = 0.002), emotional clarity (*r* = −0.37, *p* < 0.001), and emotional repair (*r* = −0.41, *p* < 0.001). In the adjusted regression model, self-esteem was the strongest variable independently associated with bullying involvement (β = −0.407, *p* < 0.001). Significant indirect effects through self-esteem were identified for emotional attention (B = −0.080, 95% CI: −0.133 to −0.027), emotional clarity (B = −0.188, 95% CI: −0.259 to −0.125), and emotional repair (B = −0.193, 95% CI: −0.270 to −0.125). **Conclusions**: School bullying involvement was associated with lower self-esteem and poorer perceived emotional competencies. Self-esteem statistically accounted for part of the associations between the three emotional intelligence dimensions and bullying involvement within the proposed exploratory models. Given the cross-sectional design, these findings should not be interpreted as evidence of causal or temporal relationships.

## 1. Introduction

School bullying is currently recognized as a major public health and educational concern due to its impact on children’s emotional well-being, psychosocial development, academic performance, and quality of life [1,2]. Bullying has been defined as a form of intentional and repeated aggression occurring within a context of power imbalance between peers, involving behaviours such as physical aggression, verbal abuse, social exclusion, intimidation, and relational violence [1]. During childhood and early adolescence, exposure to bullying situations has been associated with increased levels of anxiety, depressive symptoms, emotional dysregulation, low self-esteem, school maladjustment, and social isolation [2].

Recent international reports from the Health Behavior in School-aged Children (HBSC) study and the World Health Organization indicate that bullying remains one of the most prevalent forms of peer violence among school-aged children, with approximately 11% of adolescents reporting being bullied at school and 6% reporting bullying others across Europe [3]. Similarly, the latest Spanish HBSC report identifies bullying as a persistent public health concern, showing that peer victimization and aggressive behaviors continue to affect a considerable proportion of children and adolescents, with significant consequences for emotional well-being, school adjustment, mental health, and academic performance [4]. Research conducted in Spain has consistently identified bullying as one of the most important psychosocial challenges affecting school-aged children. Early studies highlighted the persistence of peer victimization, social exclusion, and aggressive interpersonal behaviors within Spanish schools [5]. More recently, national research has moved beyond prevalence estimates to investigate the individual, family, peer, and school-related factors associated with bullying involvement, as well as the emotional and psychosocial consequences derived from these experiences. In a systematic review focused on Primary Education, Rueda et al. concluded that bullying is a multifactorial phenomenon influenced by emotional characteristics, family environment, peer relationships, and school climate, emphasizing that effective prevention requires comprehensive interventions implemented from the earliest school years [6]. Likewise, studies conducted in Spanish Primary Education continue to report substantial levels of bullying involvement, highlighting the need to strengthen coordinated educational, psychosocial, and health-promoting strategies throughout compulsory education [7].

Beyond describing the prevalence of bullying, Spanish research has increasingly focused on understanding the emotional mechanisms underlying bullying involvement. Previous studies have shown that emotional competencies play a key role in children’s psychological adjustment and peer relationships. In a sample of Spanish adolescents, Cañas et al. [8] demonstrated that higher levels of emotional intelligence were associated with better psychological adjustment and lower peer victimization. Similarly, Rodríguez-Álvarez et al. [9] reported that stronger socio-emotional competencies were associated with a lower overlap between bullying and cyberbullying behaviors among Primary Education students. Furthermore, recent systematic reviews have confirmed that emotional intelligence consistently functions as a protective factor against bullying involvement [10,11,12]. Collectively, this growing body of Spanish evidence highlights the importance of promoting emotional competencies as a core component of comprehensive school-based bullying prevention strategies.

In recent years, growing attention has been paid to the role of emotional and psychosocial factors in explaining individual differences in bullying involvement. Among these, self-esteem and emotional intelligence have emerged as key constructs for understanding both vulnerability and resilience to peer victimization and aggressive behaviors. Self-esteem, defined as an individual’s overall evaluation of self-worth, has consistently been associated with emotional well-being, social adjustment, and adaptive interpersonal functioning during childhood. Previous evidence suggests that children with lower self-esteem are more likely to experience emotional vulnerability, poorer social relationships, and an increased risk of involvement in bullying dynamics, either as victims or aggressors [13].

Similarly, emotional intelligence, understood as the ability to perceive, understand, and regulate emotions, has been recognized as an important protective resource for children’s psychological adjustment and interpersonal functioning. Difficulties in emotional regulation and emotional understanding have been associated with maladaptive interpersonal relationships, ineffective conflict management, and increased vulnerability to school bullying [8]. Consequently, both self-esteem and emotional intelligence have been proposed as modifiable psychological resources that may contribute to preventing bullying involvement and promoting healthier peer relationships within school settings.

Bullying is currently understood as a multidimensional social phenomenon involving different participant roles, including victims, aggressors, and bystanders, whose interactions contribute to either the maintenance or prevention of bullying dynamics within school environments [14]. This complexity highlights the importance of adopting multidimensional assessment approaches capable of capturing children’s experiences across these different roles and identifying the individual and contextual factors associated with bullying involvement [9]. Such an approach is particularly relevant for designing targeted preventive interventions that address not only aggressive behaviors but also the emotional and psychosocial needs of children involved in bullying situations.

Within this framework, emotional intelligence has emerged as one of the most relevant psychological resources associated with children’s adaptation to the school environment. According to the ability model proposed by Salovey and Mayer [13], emotional intelligence refers to the capacity to perceive, understand, and regulate emotions effectively. The dimensions assessed by the Trait Meta-Mood Scale (TMMS-24)—emotional attention, emotional clarity, and emotional repair—have consistently been associated with emotional adjustment, resilience, social competence, and lower involvement in bullying behaviors among children and adolescents [8]. Recent evidence from Spanish populations has further demonstrated that emotional clarity and emotional repair are particularly important protective factors, being associated with lower levels of peer victimization, greater self-esteem, and more adaptive coping strategies [10], supporting the use of the TMMS-24 as a valuable instrument for exploring the emotional mechanisms involved in bullying prevention.

Similarly, self-esteem has consistently been identified as a key psychological resource that promotes positive self-concept, emotional well-being, resilience, and adaptive interpersonal functioning during childhood [15]. Children with higher self-esteem tend to demonstrate greater emotional security, more effective coping strategies, and healthier peer relationships, whereas low self-esteem has been associated with increased emotional vulnerability, poorer interpersonal functioning, psychosocial maladjustment, and a greater risk of involvement in bullying dynamics, particularly as victims [6]. These findings suggest that self-esteem may act not only as an indicator of children’s psychological adjustment but also as a protective resource that enhances resilience and supports positive social interactions within the school environment.

Beyond these independent associations, growing evidence suggests that the relationship between perceived emotional intelligence and bullying involvement may be explained through underlying psychological mechanisms, with self-esteem representing one of the most relevant protective resources. Emotional intelligence, particularly emotional clarity and emotional repair, may contribute to healthier peer relationships by enhancing children’s self-perceptions, emotional security, resilience, and adaptive coping strategies [8,12,13,16]. Consequently, children with greater abilities to understand and regulate their emotions are more likely to develop positive self-esteem and effective interpersonal skills, thereby reducing their vulnerability to peer victimization and bullying-related behaviors [12,14,17].

This theoretical framework is supported by previous research suggesting that self-esteem may act as a mediating mechanism through which emotional competencies exert their protective effect on bullying involvement. Although emotional intelligence and self-esteem have frequently been examined as independent protective factors, relatively few studies have explored the mechanisms linking these constructs, particularly in Spanish Primary Education populations. Investigating this potential indirect pathway may therefore provide a more comprehensive understanding of the emotional processes involved in bullying prevention and offer valuable evidence to guide the development of targeted school-based nursing interventions focused on strengthening emotional competencies and promoting children’s psychological well-being [18,19].

In parallel, social and emotional learning (SEL) interventions implemented in school settings have consistently demonstrated positive effects on emotional adjustment, interpersonal relationships, behavioral regulation, resilience, and mental health outcomes among children and adolescents [20]. Beyond improving individual emotional competencies, these interventions have also been associated with more positive school climates, reduced bullying involvement, and healthier peer relationships, highlighting the importance of promoting emotional skills from the early school years [20]. Furthermore, recent evidence suggests that strengthening socio-emotional competencies may reduce children’s vulnerability to bullying and facilitate healthier interpersonal relationships, reinforcing the value of integrating emotional education into comprehensive school-based prevention programmes [9,10,11,12,14]. Collectively, these findings support the implementation of multidisciplinary school-based interventions aimed at strengthening children’s emotional competencies as a fundamental component of comprehensive bullying prevention strategies.

Gender differences have also been widely documented in relation to emotional competencies and bullying behaviors. Evidence indicates that girls generally report higher levels of emotional awareness, emotional understanding, and emotional regulation, whereas boys tend to show higher prevalence of direct aggression, behavioral externalization, and involvement in disruptive peer interactions [21,22]. Recent Spanish studies have further suggested that these gender differences may influence not only the prevalence of bullying behaviors but also the way emotional competencies operate as protective factors against peer victimization [9]. Consequently, considering gender differences may contribute to a more comprehensive understanding of the emotional processes underlying bullying involvement during Primary Education.

Despite the growing body of international and Spanish evidence linking bullying, emotional intelligence, and self-esteem, important knowledge gaps remain. Although previous studies have consistently demonstrated independent associations between emotional intelligence, self-esteem, and bullying involvement, relatively few have examined the psychological mechanisms underlying these relationships, particularly through mediation analyses in Primary Education populations [12]. Furthermore, evidence from Spanish school settings remains limited, especially regarding studies simultaneously analyzing bullying, self-esteem, and perceived emotional intelligence from a school nursing perspective. Given the increasing role of school nurses in promoting emotional well-being and preventing school violence in Spain [23,24,25], further research is needed to better understand these relationships and to generate evidence supporting the design of more effective school-based nursing interventions.

From the perspective of school nursing, the early identification of emotional vulnerability factors constitutes a key strategy for preventing bullying and promoting emotionally healthy school environments [18,23]. School nurses are strategically positioned to implement evidence-based programmes aimed at strengthening emotional competencies, promoting self-esteem, detecting early signs of peer victimization, and coordinating multidisciplinary actions involving teachers, families, mental health professionals, and primary healthcare services [18,19].

Recent evidence from Spain has further reinforced the relevance of school nursing in bullying prevention and emotional health promotion. A nationwide study conducted within the National School Nursing Observatory demonstrated a high perceived need for school nurses among teachers, families, and educational communities, while also highlighting important territorial inequalities in their implementation [13]. Similarly, Vargas-Martínez et al. [25] reported that Spanish school nurses play a central role in health promotion, emotional education, psychosocial support, early identification of mental health problems, and coordination with schools, families, and primary healthcare professionals. These findings support the integration of school nurses into multidisciplinary strategies aimed at preventing bullying, strengthening emotional competencies, and improving children’s psychological well-being [26,27].

From a public health perspective, school-based nursing interventions focused on emotional education, resilience promotion, early detection of interpersonal conflict, and psychosocial support may contribute to improving school climate, emotional well-being, and psychosocial adjustment during critical developmental stages [18,19]. Collectively, growing international and Spanish evidence highlights school nursing as a key component of comprehensive bullying prevention programmes and supports the development of nurse-led interventions targeting children’s emotional health and healthy peer relationships.

Based on this theoretical framework, it was hypothesised that higher levels of perceived emotional intelligence would be associated with lower levels of school bullying among Primary Education students, both directly and indirectly through higher self-esteem.

Therefore, the aim of the present study was to analyse the relationships between school bullying, self-esteem, and emotional intelligence among Primary Education students from five educational centres in Madrid, examining differences according to gender and school context prior to the implementation of a nurse-led educational intervention. Additionally, this study aimed to explore whether self-esteem mediated the relationship between the dimensions of perceived emotional intelligence and school bullying involvement.

## 2. Materials and Methods

### 2.1. Study Design

A quantitative, observational, descriptive–correlational, and cross-sectional study was conducted using baseline data collected prior to the implementation of a school nursing educational intervention aimed at bullying prevention and emotional health promotion.

The study forms part of a broader quasi-experimental longitudinal research project with a pretest–posttest design developed in five primary schools located in the southern area of Madrid, Spain. For confidentiality purposes, participating schools were anonymized and identified as Centre 1, Centre 2, Centre 3, Centre 4, and Centre 5.

Because the present analyses were performed exclusively using baseline data collected before the intervention, all associations and mediation analyses should be interpreted as exploratory cross-sectional findings and not as evidence of causal or temporal relationships.

The study was reported in accordance with the Strengthening the Reporting of Observational Studies in Epidemiology (STROBE) guidelines for cross-sectional studies.

### 2.2. Study Population

The study population consisted of Primary Education students enrolled in the 4th, 5th, and 6th grades from five participating educational centres located in Madrid, Spain. A total of 507 students participated in the study, with a mean age of 10.08 years (*SD* = 0.82). The sample presented a balanced gender distribution, comprising 50.7% female students and 46.2% male students; 3.1% of participants did not report their gender or selected another category.

The sample represents children in late childhood, a developmental stage characterised by substantial emotional, social, and interpersonal development and considered particularly relevant for the emergence of bullying behaviours and emotional vulnerability.

The participating schools included both public and state-subsidised (concerted) educational centres serving urban populations in the southern metropolitan area of Madrid, Spain. All schools provided compulsory Primary Education and enrolled students from the 4th, 5th, and 6th grades, corresponding to the target population of the educational intervention. Although the schools differed in the number of enrolled students, they were classified as medium-to-large educational centres and shared similar socioeconomic characteristics within their catchment areas. To preserve institutional confidentiality and comply with the agreements established with the participating schools, detailed information regarding school names, catchment areas, and other institutional characteristics that could permit school identification has not been reported. Nevertheless, all participating centres operated under the Spanish national educational framework and provided comparable educational environments for the purposes of the present study.

### 2.3. Inclusion Criteria

Students were eligible for participation if they met all of the following criteria: (1) enrolment in one of the participating educational centers; (2) attendance in the 4th, 5th, or 6th grades of Primary Education; (3) provision of written informed consent by a parent or legal guardian; and (4) willingness to participate in the study.

### 2.4. Sample Size and Participants

Participants were recruited through non-probabilistic convenience sampling, based on the accessibility and voluntary participation of the educational centres involved in the intervention programme. All eligible students from the participating schools who met the inclusion criteria and provided the required consent were invited to participate. A total of 507 students completed the study questionnaires and constituted the final analytical sample.

To justify the adequacy of the sample size, a statistical power analysis was considered for the main correlational analyses. Assuming a small-to-moderate effect size (*r* = 0.15), a two-tailed significance level of *α* = 0.05, and statistical power of 0.80, the minimum required sample size was approximately 346 participants. Therefore, the final sample of 507 students exceeded the minimum required sample size and was considered sufficient to detect small-to-moderate associations between school bullying, self-esteem, and perceived emotional intelligence.

Questionnaires with incomplete responses or missing data affecting one or more of the variables included in the regression or mediation analyses were excluded from those specific analyses using listwise deletion. Consequently, the number of participants included varied slightly depending on the statistical procedure performed, and the corresponding sample size is reported for each analysis where appropriate.

### 2.5. Ethical Procedures

The study was conducted in accordance with the ethical principles established in the Declaration of Helsinki and current regulations governing research involving minors and the protection of personal data.

Participation was voluntary, anonymous, and confidential. Written informed consent was obtained from parents or legal guardians prior to data collection, and verbal assent was obtained from all participating students.

The study protocol was approved by the Research Ethics Committee for Human Subjects of Universitat Jaume I (Reference: CEISH/08/2025; approval date: 12 May 2025).

Data confidentiality and participant privacy were guaranteed throughout all phases of the study in accordance with the General Data Protection Regulation (GDPR) and Spanish Organic Law 3/2018 on Personal Data Protection and Guarantee of Digital Rights.

### 2.6. Instruments

Three validated psychometric instruments were used to assess school bullying involvement, self-esteem, and perceived emotional intelligence among the participating students.

#### 2.6.1. Peer Harassment Questionnaire (PHQ)

School bullying involvement was assessed using the Peer Harassment Questionnaire (PHQ), a validated instrument designed to evaluate experiences related to peer victimization, aggression, and bullying involvement in school settings [14].

The PHQ comprises 39 items grouped into eight dimensions assessing different manifestations of peer harassment, including verbal abuse, direct social exclusion, threats, cyberbullying, indirect social exclusion, object-based aggression, physical abuse, and aggressive behaviours. Responses are recorded on a Likert-type scale ranging from “never” to “always”, and item scores are summed to obtain a global score, with higher scores indicating greater involvement in bullying-related situations. Although the questionnaire primarily evaluates experiences associated with peer victimization, it also includes dimensions related to aggressive behaviours and overall exposure to bullying situations. Therefore, in the present study, the total score was interpreted as an indicator of overall involvement in school bullying, consistent with previous research using this instrument [9,14].

For descriptive purposes, bullying involvement levels (low, moderate, and high) were established according to the distribution of total scores in the study sample. These categories were used exclusively for descriptive analyses, whereas all inferential analyses were performed using the continuous total score.

Previous validation studies conducted in Spanish child and adolescent populations have demonstrated an eight-factor structure, satisfactory construct validity, high internal consistency, and good reliability, supporting its use for assessing bullying involvement in school settings [9,14]. Cronbach’s α for the PHQ in the present study was 0.90, indicating excellent internal consistency.

#### 2.6.2. Self-Esteem Questionnaire for Primary Education (A-EP)

Self-esteem was evaluated using the Self-Esteem Questionnaire for Primary Education (A-EP), a psychometric instrument specifically developed and validated for Primary Education students [14].

The A-EP consists of 17 dichotomous items (Yes/No) designed to assess children’s global self-esteem in educational contexts. Total scores range from lower to higher levels of perceived self-esteem, with higher scores reflecting a more positive self-concept.

The scale assesses students’ self-perception and emotional self-concept through a global self-esteem score, with higher scores indicating more positive levels of self-esteem.

For interpretative purposes, self-esteem scores were classified as low, moderate, or high.

Previous validation studies conducted in Spanish Primary Education populations have demonstrated adequate construct validity, satisfactory internal consistency, and good reliability, supporting its use for assessing global self-esteem in school-aged children [14]. Cronbach’s α for the A-EP in the present study was 0.85, indicating good internal consistency.

#### 2.6.3. Trait Meta-Mood Scale (TMMS-24)

Perceived emotional intelligence was assessed using the Trait Meta-Mood Scale (TMMS-24), a validated self-report instrument composed of three dimensions: Emotional Attention, Emotional Clarity, and Emotional Repair [5].

Emotional Attention refers to the awareness and monitoring of emotions, Emotional Clarity assesses emotional understanding, and Emotional Repair evaluates the ability to regulate and manage emotional states.

Higher scores indicate better perceived emotional competencies.

The TMMS-24 consists of 24 items, with eight items for each dimension, rated on a five-point Likert scale ranging from 1 (“strongly disagree”) to 5 (“strongly agree”). Scores are calculated separately for each dimension, as recommended by the instrument developers.

The Spanish adaptation of the TMMS-24 has consistently demonstrated a stable three-factor structure, adequate construct validity, and high internal consistency across child, adolescent, and young adult populations. More recent studies conducted in Spain have further confirmed its psychometric robustness and suitability for assessing perceived emotional intelligence in educational settings [5,10].

Cronbach’s α coefficients obtained in the present study were 0.90 for Emotional Attention, 0.90 for Emotional Clarity, and 0.86 for Emotional Repair, indicating good-to-excellent internal consistency across all dimensions.

### 2.7. Data Collection Procedure

Data collection was conducted collectively within the participating schools during regular school hours between October 2025 and January 2026.

Questionnaires were administered in classroom settings under the supervision of the research team to ensure standardized administration procedures and adequate comprehension of the instructions. Participants completed the questionnaires anonymously using coded identification numbers, thereby guaranteeing confidentiality and preventing personal identification.

Prior to data collection, authorization was obtained from the participating school administrations and teaching staff. Parents or legal guardians provided written informed consent, and all students were informed about the voluntary, anonymous, and confidential nature of the study before completing the questionnaires.

As the present study reports baseline findings obtained before implementation of the educational intervention, all questionnaires were administered prior to any training or preventive activities conducted by the research team.

### 2.8. Statistical Analysis

Statistical analyses were performed using IBM SPSS Statistics version 29.0 (IBM Corp., Armonk, NY, USA).

Descriptive statistics were calculated for all study variables. Categorical variables were expressed as frequencies and percentages, whereas continuous variables were summarized using means, standard deviations, minimum values, and maximum values. Preliminary analyses showed no statistically significant differences in bullying scores between educational centres; therefore, school was not included as a covariate in the regression and mediation models.

The normality of continuous variables was assessed using the Shapiro–Wilk test. Comparisons according to sex and educational centre were performed using Pearson’s chi-square test for categorical variables and Student’s *t*-test or the Mann–Whitney U test for continuous variables, as appropriate.

Effect sizes were calculated using Cohen’s d for continuous variables and Cramer’s V for categorical variables and interpreted according to conventional criteria proposed by Cohen.

Associations between school bullying, self-esteem, and the dimensions of perceived emotional intelligence were examined using Pearson’s correlation coefficient (*r*). Correlation strength was interpreted according to established psychometric guidelines.

Additionally, a multiple linear regression analysis was performed to identify the variables independently associated with school bullying. The total school bullying score was entered as the dependent variable, whereas self-esteem, emotional attention, emotional clarity, emotional repair, and sex were included as independent variables. Regression assumptions, including normality of residuals, homoscedasticity, independence of errors (Durbin–Watson statistic), and multicollinearity (Variance Inflation Factor and tolerance values), were assessed before model estimation. Standardized regression coefficients (*β*), 95% confidence intervals (95% *CI*), and the coefficient of determination (*R*^2^) were reported.

To further explore the potential indirect statistical associations between perceived emotional intelligence and school bullying involvement, an exploratory mediation analysis was conducted according to the proposed theoretical framework. The mediation model examined whether self-esteem statistically accounted for part of the association between perceived emotional intelligence dimensions and school bullying involvement.

Mediation analyses were performed using the PROCESS macro for SPSS (Model 4; Hayes). The three perceived emotional intelligence dimensions evaluated by the TMMS-24 (emotional attention, emotional clarity, and emotional repair) were considered independent variables, self-esteem was included as the statistical mediator, and the total school bullying score was entered as the dependent variable. Gender was incorporated as a covariate due to previously reported differences in emotional competencies and bullying-related behaviours.

Indirect effects were estimated using a non-parametric bootstrapping procedure with 5000 resamples and 95% bias-corrected confidence intervals (*CIs*). The indirect mediation effect was considered statistically significant when the confidence interval did not include zero.

All statistical tests were two-tailed, and statistical significance was established at *p* < 0.05.

## 3. Results

A total of 507 Primary Education students from five educational centers in Madrid participated in the study. The mean age of the sample was 10.08 years (SD = 0.82), and participants were relatively evenly distributed across the 4th, 5th, and 6th grades. Female students represented 50.7% of the sample, male students 46.2%, and 3.1% either did not report their sex or selected a category other than female or male. These participants were not included in sex-based comparative analyses due to the limited number of cases.

Baseline self-esteem scores indicated moderate-to-high levels of self-esteem within the overall sample (M = 26.61, SD = 4.38). Descriptive characteristics according to educational center, including age, sex distribution, educational grade, and mean self-esteem scores, are presented in Table 1.

Table 2 presents the internal consistency reliability coefficients of the instruments administered in the study. All scales demonstrated good-to-excellent reliability, with Cronbach’s alpha values ranging from 0.85 to 0.90, supporting the adequacy of the instruments for assessing school bullying, self-esteem, and perceived emotional intelligence in the study population.

Table 3 presents the distribution of school bullying levels across the five participating educational centers at pretest. Moderate bullying was the most prevalent category in the overall sample, accounting for 51.3% of participants, whereas 35.9% and 12.8% were classified as having low and high bullying levels, respectively. Although slight variations were observed in the distribution of bullying levels across educational centers, moderate bullying remained the predominant category in all schools. Comparative analysis showed no statistically significant differences according to educational center (Pearson’s χ^2^ = 8.38, *p* = 0.397; Cramer’s V = 0.09), indicating a negligible association between school context and bullying level. Bullying involvement levels were categorized according to the scoring criteria established for the Peer Harassment Questionnaire (PHQ), with participants classified into low, moderate, or high involvement groups based on the corresponding cutoff points of the instrument.

Table 4 presents the distribution of self-esteem levels according to sex across the five participating educational centres at pretest. Moderate self-esteem was the most prevalent category in both sexes, accounting for 59.8% of male students and 56.0% of female students.

Female students consistently showed higher proportions of high self-esteem and lower proportions of low self-esteem than male students across the participating educational centres. Overall, these differences were statistically significant (*p* = 0.022; Cramer’s V = 0.18), indicating a small but meaningful association between sex and self-esteem level.

Table 5 presents the distribution of school bullying levels according to sex across the five participating educational centres at pretest. Moderate bullying was the most prevalent category in both sexes, accounting for 53.8% of male students and 49.0% of female students.

Male students consistently showed higher proportions of high bullying levels and lower proportions of low bullying levels than female students across the participating educational centres. Overall, these differences were statistically significant (*p* = 0.009; Cramer’s V = 0.22), indicating a small-to-moderate association between sex and school bullying level.

Table 6 presents the TMMS-24 emotional intelligence dimensions according to sex across the five participating educational centres at pretest. Female students consistently obtained higher mean scores than male students in emotional attention, emotional clarity, and emotional repair.

Overall, significant differences between sexes were observed in emotional intelligence dimensions (*p* = 0.012), with a moderate effect size (Cohen’s d = 0.52). Female students reported higher perceived emotional competencies than male students across the participating educational centres. This pattern was observed across the three emotional intelligence dimensions evaluated by the TMMS-24.

**Figure 1:**
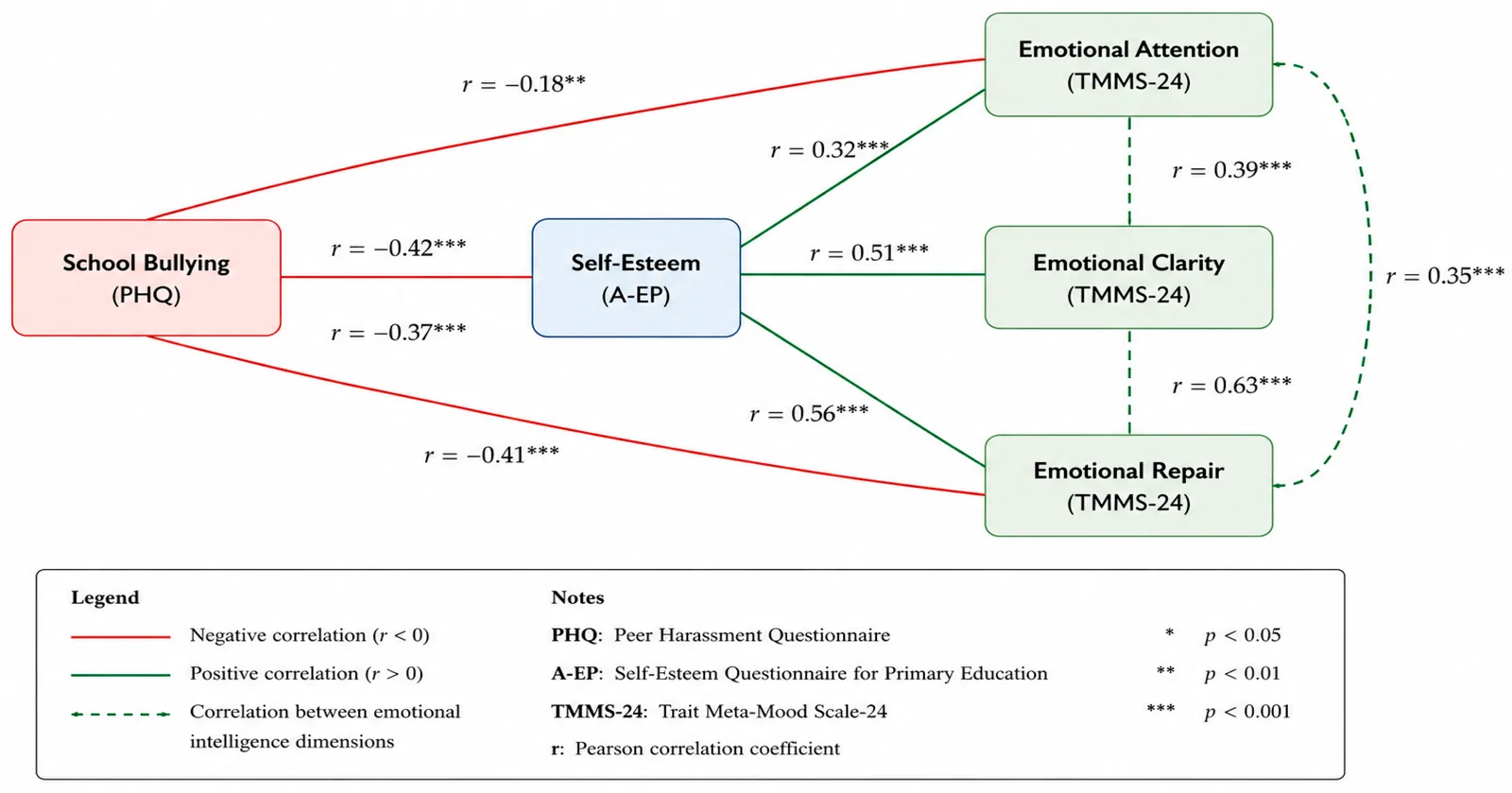
Correlation network between school Bullying, self-esteem, and perceived emotional intelligence dimension at pretest.

Table 7 presents the correlations between school bullying, self-esteem, and the emotional intelligence dimensions assessed by the TMMS-24 at pretest. School bullying showed significant negative correlations with self-esteem (r = −0.42, *p* < 0.001), emotional attention (r = −0.18, *p* = 0.002), emotional clarity (r = −0.37, *p* < 0.001), and emotional repair (r = −0.41, *p* < 0.001). Conversely, self-esteem was positively correlated with emotional attention (r = 0.32, *p* < 0.001), emotional clarity (r = 0.51, *p* < 0.001), and emotional repair (r = 0.56, *p* < 0.001). Significant positive associations were also observed among the emotional intelligence dimensions, with the strongest correlation identified between emotional clarity and emotional repair (r = 0.63, *p* < 0.001). Figure 1 illustrates the correlation network between school bullying, self-esteem, and perceived emotional intelligence dimensions at pretest.

Table 8 presents the results of the multiple linear regression analysis performed to identify the variables independently associated with school bullying. The analysis was conducted using complete-case analysis, including the 411 participants with complete data for all variables entered into the model. Lower self-esteem emerged as the strongest independent predictor of higher school bullying scores (β = −0.417, *p* < 0.001). Emotional attention showed a weak positive association with school bullying, whereas emotional clarity, emotional repair, and sex were not statistically significant predictors after adjustment. The regression model explained 17.2% of the variance in school bullying (Adjusted *R*^2^ = 0.162), indicating that self-esteem plays a central role in the observed associations with school bullying in this population and supports its evaluation as a potential statistical mediator within the proposed theoretical model.

Table 9 presents the results of the exploratory mediation analyses examining the indirect effects of the three perceived emotional intelligence dimensions on school bullying involvement through self-esteem; An exploratory mediation analysis was conducted to examine the statistical indirect association between emotional repair and school bullying through self-esteem. Emotional repair was positively associated with self-esteem (path a: B = 0.192, *p* < 0.001), indicating that students with greater perceived ability to regulate their emotions also reported higher levels of self-esteem. In turn, self-esteem was negatively associated with school bullying involvement (path b: B = −0.960, *p* < 0.001).

The indirect effect of emotional repair on school bullying through self-esteem was statistically significant (B = −0.184, SE = 0.043, 95% CI: −0.276 to −0.108). After accounting for self-esteem, the direct effect of emotional repair on school bullying was not statistically significant (B = 0.095, *p* = 0.159). These findings suggest that self-esteem statistically accounted for part of the observed association between emotional repair and school bullying within the proposed exploratory mediation model.

Given the cross-sectional design of the present study, these findings should be interpreted as exploratory statistical associations and do not establish causal or temporal relationships.

## 4. Discussion

The present study examined the relationships between school bullying, self-esteem, and perceived emotional intelligence among Primary Education students from five educational centers in Madrid prior to the implementation of a nurse-led educational intervention. Four principal findings emerged. First, moderate bullying involvement was the most frequently observed category across the participating schools, with no statistically significant differences according to educational center. Second, female students consistently reported higher levels of self-esteem and perceived emotional intelligence than male students. Third, school bullying was significantly associated with lower self-esteem and poorer emotional competencies, particularly emotional clarity and emotional repair. Finally, the mediation analysis identified self-esteem as a significant indirect pathway within the proposed theoretical model, suggesting that self-esteem may statistically account for part of the observed association between emotional repair and school bullying involvement. However, given the cross-sectional design of the study, these findings should be interpreted as exploratory statistical associations rather than evidence of causal or protective mechanisms. Overall, these findings reinforce the importance of considering emotional competencies and self-esteem as key psychosocial factors associated with bullying involvement during Primary Education and support the development of comprehensive school-based prevention strategies from a school nursing perspective.

Moderate levels of bullying involvement were identified across the sample, with approximately one in eight students classified as presenting high bullying involvement according to the classification criteria applied in this study. These findings are consistent with previous studies indicating that bullying involvement remains a relevant psychosocial concern during late childhood and preadolescence [1,2]. Recent Spanish studies have similarly reported that peer victimization, social exclusion, and aggressive interpersonal behaviors continue to affect a substantial proportion of Primary Education students, highlighting the persistence of bullying across different educational settings despite increasing prevention efforts [23]. These findings are also consistent with the most recent Spanish HBSC report, which identifies bullying as one of the principal threats to children’s emotional well-being and school adjustment [4].

One of the principal findings of the study was the significant inverse association between bullying involvement and emotional adjustment variables. Students presenting higher bullying scores reported lower levels of self-esteem, emotional attention, emotional clarity, and emotional repair. These findings are consistent with previous research suggesting that emotional dysregulation, difficulties in emotional understanding, and negative self-perception are associated with both peer victimization and aggressive interpersonal behaviors during childhood and adolescence [5,6,7,8,9]. Our findings are also in agreement with recent Spanish studies reporting that higher levels of perceived emotional intelligence, particularly emotional clarity and emotional repair, are associated with better psychological adjustment, greater social competence, and lower involvement in bullying behaviors [28,29]. Furthermore, recent systematic reviews have consistently identified emotional intelligence as an important protective factor against bullying involvement, highlighting emotional regulation as a key target for school-based preventive interventions [10,12].

Particularly relevant was the association observed between bullying involvement and emotional repair. Students with lower perceived ability to regulate and manage their emotions reported greater involvement in bullying-related situations. These findings are consistent with previous research indicating that difficulties in emotional regulation are associated with maladaptive coping responses, interpersonal conflict, impulsive behaviors, and poorer psychosocial adaptation in school environments [14]. Likewise, the strongest positive correlations were identified between emotional clarity and emotional repair (r = 0.63) and between self-esteem and emotional repair (r = 0.56), indicating that these psychological dimensions are closely interrelated. However, these associations should not be interpreted as evidence of a direct protective effect against bullying involvement.

Similarly, self-esteem demonstrated moderate-to-strong positive correlations with all dimensions of perceived emotional intelligence, particularly emotional clarity and emotional repair. These findings are consistent with previous evidence suggesting that emotional competencies are associated with psychological well-being, social adaptation, and healthy interpersonal functioning in school-aged children [13,28]. Within the present study, these findings should be interpreted as evidence of statistical associations among these emotional constructs rather than as confirmation of protective pathways or causal relationships with bullying involvement.

Beyond these associations, the exploratory mediation analysis provides additional information regarding the statistical relationships among emotional competencies, self-esteem, and bullying involvement. The findings showed a significant indirect effect of emotional repair on bullying involvement through self-esteem within the proposed theoretical model, suggesting that self-esteem may statistically account for part of the observed association between emotional regulation abilities and bullying involvement. This finding is consistent with previous theoretical and empirical research suggesting that self-esteem may represent an important psychological mechanism linking emotional competencies with children’s social adjustment and emotional well-being, although evidence specifically examining mediation pathways in Primary Education remains limited [12,16]. Children with greater perceived ability to regulate emotional states also reported higher self-esteem; however, the cross-sectional nature of the baseline data precludes establishing the temporal order or directionality of these associations.

Sex-related differences represent another important finding of the study. Female students consistently reported higher levels of self-esteem and perceived emotional intelligence, whereas male students showed higher bullying involvement scores and lower scores in some emotional variables [12,29]. These results are consistent with the previous literature reporting sex-related differences in emotional competencies during childhood and adolescence [8,15]. Similar patterns have also been reported in Spanish school populations, where girls generally demonstrate higher emotional awareness and emotional regulation, whereas boys tend to report greater involvement in direct forms of aggression and bullying behaviors, although these differences are influenced by developmental and contextual factors [15,30]. However, the present study did not directly assess externalizing behaviors, aggression patterns, or behavioral dysregulation; therefore, these characteristics cannot be inferred from the current findings. The observed sex-related differences may reflect a combination of developmental, social, and educational factors previously described in the literature, although these potential explanations were not specifically examined in the present study. Consequently, future bullying prevention programmes may consider adopting gender-sensitive approaches that address diverse emotional and relational needs within school settings.

**Implications for Nursing Practice**

The present findings highlight the potential role of school nurses in the early identification of bullying-related emotional vulnerability among Primary Education students [18,19]. The significant associations identified between bullying involvement, lower self-esteem, and poorer emotional competencies suggest that routine emotional and psychosocial screening within school settings may facilitate the early identification of students who could benefit from additional assessment or support. Incorporating validated emotional assessment tools into school health programmes may assist in planning preventive and individualized nursing interventions focused on emotional well-being and psychosocial resilience [18]. Recent evidence from the Spanish National School Nursing Observatory has also highlighted the growing role of school nurses in promoting mental health, identifying emotional and psychosocial needs, coordinating multidisciplinary care, and implementing preventive strategies within educational settings [24,25]. These findings reinforce the relevance of integrating emotional assessment into routine school nursing practice.

Furthermore, school nurses may contribute to bullying prevention through the implementation of psychoeducational interventions aimed at promoting emotional competencies, emotional regulation, self-esteem, and adaptive conflict resolution skills [19,20]. The exploratory mediation analysis suggests that self-esteem may statistically account for part of the observed association between emotional competencies and bullying involvement within the proposed theoretical model. Although these findings may help inform the design of future nurse-led emotional health programmes, the present study did not directly evaluate the effectiveness of such interventions. Therefore, their impact on reducing bullying involvement and improving psychosocial outcomes should be examined in future longitudinal and intervention studies [19]. These findings are consistent with recent systematic reviews indicating that school-based nursing interventions focused on emotional competencies and psychosocial support may contribute to improving emotional well-being, school climate, and bullying prevention, although further high-quality intervention studies remain necessary to establish their effectiveness [19,20].

These interventions may be particularly relevant during late childhood and preadolescence, a developmental stage characterized by increased emotional sensitivity and vulnerability to peer-group dynamics.

The present findings also highlight the importance of interdisciplinary collaboration among school nurses, teachers, psychologists, families, and educational administrators in addressing bullying from a comprehensive and preventive perspective. Comprehensive school mental health approaches integrating emotional health promotion, early psychosocial support, and school climate improvement strategies may contribute to creating safer and more emotionally supportive educational environments [20,26]. Consequently, the integration of school nursing into multidisciplinary mental health and bullying prevention frameworks may represent a valuable public health strategy that warrants further evaluation in educational settings.

The present findings, together with the growing body of national and international evidence, support the integration of school nurses within multidisciplinary frameworks for bullying prevention, emotional health promotion, and early psychosocial intervention. Strengthening the presence of school nurses in educational settings may therefore represent an important public health strategy for improving children’s emotional well-being, fostering safer school environments, and promoting the early identification and support of students at risk [24,25].

**Strengths and Limitations**

This study presents several methodological strengths. First, its multicenter design and the inclusion of students from different educational contexts provide a broader perspective on the psychosocial characteristics of the participating school populations. Second, the simultaneous assessment of school bullying, self-esteem, and perceived emotional intelligence using validated psychometric instruments with high internal consistency provides a comprehensive multidimensional perspective on psychosocial functioning during childhood. Third, the incorporation of effect size measures, predictive regression models, and exploratory mediation analyses strengthens the robustness and interpretability of the findings by examining the statistical associations among emotional competencies, self-esteem, and bullying involvement. Finally, the sex-based analytical approach contributes to a more comprehensive understanding of emotional and relational characteristics in Primary Education populations.

Nevertheless, several limitations should be acknowledged. The cross-sectional nature of this pretest analysis does not allow causal relationships between variables to be established. Therefore, although the mediation analyses were conducted within a theoretically specified framework, longitudinal studies are needed to clarify the temporal relationships and the directionality of the observed associations. In addition, the use of self-report measures may have introduced social desirability and response biases. Although the sample size was substantial, participants were recruited through convenience sampling from a limited geographical area, which may reduce the generalizability of the findings to other educational contexts or populations.

Future longitudinal analyses derived from post-intervention and follow-up assessments will be necessary to evaluate the effects of the nurse-led educational programme and to determine whether changes in emotional competencies and self-esteem are associated with changes in bullying involvement over time. Further research should also explore additional mediating and moderating factors, including school climate, peer relationships, family environment, and contextual variables, to provide a more comprehensive understanding of the factors associated with school bullying.

Despite these limitations, the present findings contribute to the growing evidence regarding the emotional and psychosocial factors associated with school bullying among Primary Education students. They also highlight the potential value of preventive strategies focused on emotional health promotion within school communities. Although the present study did not evaluate the effectiveness of school nursing interventions, the findings may help inform the development of integrated, school-based mental health initiatives aimed at promoting psychosocial well-being, strengthening emotional competencies, and supporting future bullying prevention efforts during childhood [25,26].

## 5. Conclusions

The present study identified significant associations between school bullying involvement, self-esteem, and perceived emotional intelligence among Primary Education students, highlighting the relevance of emotional competencies in relation to children’s psychosocial well-being. Students with higher emotional clarity, emotional repair, and self-esteem reported lower bullying involvement, highlighting the close interrelationship among emotional health, self-esteem, and peer relationships within the school environment.

Furthermore, the regression and exploratory mediation analyses provided additional insight into the statistical associations among emotional competencies, self-esteem, and bullying involvement. Self-esteem emerged as the strongest independent variable associated with school bullying and statistically accounted for part of the observed association between emotional repair and bullying involvement within the proposed theoretical model. However, given the cross-sectional design of the study, these findings should be interpreted as exploratory statistical associations and do not establish causal or temporal relationships.

Significant sex-related differences were identified, with female students reporting higher levels of self-esteem and perceived emotional intelligence and male students showing higher bullying involvement scores. These findings highlight the importance of considering sex-related differences when designing preventive strategies that address the emotional and relational needs of children during late childhood and preadolescence.

From a school nursing perspective, the findings suggest that early emotional assessment and psychoeducational interventions targeting emotional competencies and self-esteem may represent valuable components of school health promotion programmes. However, the present study did not directly evaluate the effectiveness of nurse-led interventions, and their impact on bullying prevention should be investigated in future longitudinal and intervention studies.

Overall, this study contributes to the growing evidence on the relationships among emotional competencies, self-esteem, and school bullying in Primary Education. Future longitudinal and intervention research is warranted to clarify the temporal relationships among these variables and to evaluate the effectiveness of school-based emotional health programmes aimed at promoting children’s psychosocial well-being.

## Figures and Tables

**Table 1:** Descriptive characteristics of the study population by educational center.

Educational Centre	*n*	Mean Age (SD)	4th Grade/*n* (%)	5th Grade/*n* (%)	6th Grade/*n* (%)	Mean Self-Esteem (SD)
Centre 1	149	10.26 (0.91)	46 (30.9)	55 (36.9)	48 (32.2)	26.20 (4.72)
Centre 2	40	10.72 (0.60)	10 (25.0)	14 (35.0)	16 (40.0)	26.30 (4.93)
Centre 3	90	9.86 (0.76)	32 (35.6)	30 (33.3)	28 (31.1)	27.53 (3.80)
Centre 4	125	9.90 (0.85)	44 (35.2)	42 (33.6)	39 (31.2)	26.62 (4.36)
Centre 5	103	9.87 (0.64)	36 (35.0)	35 (34.0)	32 (31.0)	26.62 (4.42)
Total	507	10.08 (0.82)	168 (33.1)	176 (34.7)	163 (32.2)	26.61 (4.38)

Abbreviation: SD, standard deviation; *n*, number of participants.

**Table 2:** Internal consistency reliability coefficients of the study instruments.

Instrument	Cronbach’s α
Peer Harassment Questionnaire (PHQ)	0.90
Self-Esteem Questionnaire for Primary Education (A-EP)	0.85
TMMS-24 Emotional Attention	0.90
TMMS-24 Emotional Clarity	0.90
TMMS-24 Emotional Repair	0.86

Abbreviation: α, Cronbach’s alpha.

**Table 3:** Distribution of school bullying levels according to educational center at pretest.

Educational Centre	Low/*n* (%)	Moderate/ *n* (%)	High/*n* (%)	Total/ *n*	*p*-Value	Cramer’s V
Centre 1	45 (30.2)	82 (55.0)	22(14.8)	149	0.397	0.09
Centre 2	13 (32.5)	21(52.5)	6 (15.0)	40		
Centre 3	39 (43.3)	42 (46.7)	9 (10.0)	90		
Centre 4	40 (32.0)	68 (54.4)	17 (13.6)	125		
Centre 5	45 (43.7)	47 (45.6)	11 (10.7)	103		
Total	182(35.9)	260 (51.3)	65 (12.8)	507	0.397	0.09

Note: *p* < 0.05.

**Table 4:** Distribution of self-esteem levels according to sex and educational center at pretest.

Educational Centre	Sex	Low Self-Esteem/*n* (%)	Moderate Self-Esteem/*n* (%)	High Self-Esteem/*n* (%)	Total/ *n*	*p*-Value	Cramer’s V
Centre 1	Female	9 (11.8)	45 (59.2)	22 (29.0)	76	0.031	0.21
	Male	13 (22.8)	33 (57.9)	11 (19.3)	57		
Centre 2	Female	3 (13.0)	12 (52.2)	8 (34.8)	23	0.118	0.16
	Male	4 (23.5)	10 (58.8)	3 (17.7)	17		
Centre 3	Female	2 (4.7)	21 (48.8)	20 (46.5)	43	0.042	0.19
	Male	5 (10.6)	30 (63.8)	12 (25.6)	47		
Centre 4	Female	5 (8.8)	33 (57.9)	19 (33.3)	57	0.084	0.13
	Male	11 (16.2)	40 (58.8)	17 (25.0)	68		
Centre 5	Female	5 (8.6)	33 (56.9)	20 (34.5)	58	0.091	0.12
	Male	8 (17.8)	27 (60.0)	10 (22.2)	45		
Total	Female	24 (9.3)	144 (56.0)	89 (34.7)	257	0.022	0.18
	Male	41 (17.5)	140 (59.8)	53 (22.7)	234		

Abbreviations: *n*, number of participants; Cramer’s V, effect size measure for chi-square tests.

**Table 5:** Distribution of school bullying levels according to sex and educational center at pretest.

Educational Centre	Sex	Low/*n* (%)	Moderate/*n* (%)	High/*n* (%)	Total/*n*	*p*-Value	Cramer’s V
Centre 1	Female	28 (36.8)	41 (53.9)	7 (9.3)	76	0.018	0.24
	Male	14 (24.6)	31 (54.4)	12 (21.0)	57		
Centre 2	Female	9 (39.1)	11 (47.8)	3 (13.1)	23	0.094	0.17
	Male	4(23.5)	9 (52.9)	4 (23.6)	17		
Centre 3	Female	9 (39.1)	19 (44.2)	2 (4.6)	43	0.041	0.20
	Male	16 (34.0)	24 (51.1)	7 (14.9)	47		
Centre 4	Female	22 (51.2)	31(54.4)	5 (8.8)	57	0.037	0.19
	Male	18 (26.5)	38 (55.9)	12 (17.6)	68		
Centre 5	Female	30 (51.7)	24 (41.4)	4 (6.9)	58	0.049	0.18
	Male	13 (28.9)	24 (53.3)	8 (17.8)	45		
Total	Female	110 (42.8)	126 (49.0)	21 (8.2)	257	0.009	0.22
	Male	65 (27.8)	126 (53.8)	43 (18.4)	234		

**Table 6:** TMMS-24 emotional intelligence dimensions according to sex and educational center at pretest.

Educational Centre	Sex	Low, *n* (%)	Moderate, *n* (%)	High, *n* (%)	Total, *n*	*p*-Value	Cramer’s V
Centre 1	Female	27 (4.7)	25.3 (4.5)	24.8 (4.6)	76	0.031	0.54
	Male	24.3(5.1)	22.1 (4.8)	21.4 (5.0)	57		
Centre 2	Female	26.1(4.9)	24.8 (4.2)	23.6 (4.5)	23	0.084	0.31
	Male	23.8(5.3)	21.7 (5.0)	20.9 (4.8)	17		
Centre 3	Female	28.1(4.2)	26.0 (4.0)	25.1 (4.1)	43	0.018	0.63
	Male	24.7(4.8)	22.9 (4.3)	22.0 (4.6)	47		
Centre 4	Female	27.2(4.6)	25.1 (4.1)	24.4 (4.2)	57	0.027	0.49
	Male	24.1(5.0)	22.3 (4.6)	21.7 (4.8)	68		
Centre 5	Female	27.4(4.3)	25.5 (4.0)	24.9 (4.1)	58	0.049	0.45
	Male	24.5(4.9)	22.4 (4.5)	21.9 (4.7)	45		
Total	Female	27.3(4.5)	25.3 (4.2)	24.6 (4.3)	257	0.012	0.52
	Male	24.3(5.0)	22.3 (4.6)	21.6 (4.8)	234		

**Table 7:** Correlations between school bullying, self-esteem, and emotional intelligence dimensions at pretest.

Variables	Pearson’s r	95% CI	*p*-Value
School Bullying—Self-Esteem	−0.42	−0.49 to −0.34	<0.001
School Bullying—Emotional Attention	−0.18	−0.26 to −0.09	0.002
School Bullying—Emotional Clarity	−0.37	−0.44 to −0.29	<0.001
School Bullying—Emotional Repair	−0.41	−0.48 to −0.33	<0.001
Self-Esteem—Emotional Attention	0.32	0.24 to 0.40	<0.001
Self-Esteem—Emotional Clarity	0.51	0.44 to 0.58	<0.001
Self-Esteem—Emotional Repair	0.56	0.49 to 0.62	<0.001
Emotional Attention—Emotional Clarity	0.39	0.31 to 0.46	<0.001
Emotional Attention—Emotional Repair	0.35	0.27 to 0.43	<0.001
Emotional Clarity—Emotional Repair	0.63	0.57 to 0.69	<0.001

**Table 8:** Multiple linear regression analysis identifying factors independently associated with school bullying based on participants with complete data.

Predictor	B	SE	β	95% CI for B	*p*-Value
Constant	33.509	2.830	-	27.947 to 39.071	<0.001
Self-esteem	−0.936	0.106	−0.407	−1.143 to −0.728	<0.001
Emotional Attention	0.308	0.075	0.240	0.160 to 0.455	<0.001
Emotional Clarity	−0.278	0.085	−0.222	−0.445 to −0.111	0.001
Emotional Repair	0.054	0.081	0.043	−0.106 to 0.214	0.506
Sex (girls vs. boys)	−3.025	0.863	−0.149	−4.720 to −1.329	0.001

Model fit: F(5405) = 16.81; *p* < 0.001; R^2^ = 0.172; Adjusted R^2^ = 0.162; Durbin–Watson = 1.61. Abbreviations: B, unstandardized regression coefficient; SE, standard error; β, standardized regression coefficient; CI, confidence interval.

**Table 9:** Mediation analysis examining the indirect effect of emotional repair on school bullying involvement through self-esteem.

TMMS-24 Dimension	Pathway	B	SE	95% CI	*p*-Value
Emotional Attention	Path a: Attention → Self-esteem	0.076	0.027	0.024 to 0.129	0.004
Emotional Attention	Path b: Self-esteem → Bullying	−1.047	0.099	−1.241 to −0.853	<0.001
Emotional Attention	Total effect (c)	0.083	0.061	−0.038 to 0.204	0.178
Emotional Attention	Direct effect (c′)	0.163	0.055	0.054 to 0.271	0.003
Emotional Attention	Indirect effect (a × b)	−0.080	0.027	−0.133 to −0.027	—
Emotional Clarity	Path a: Clarity → Self-esteem	0.191	0.024	0.143 to 0.239	<0.001
Emotional Clarity	Path b: Self-esteem → Bullying	−0.985	0.105	−1.192 to −0.778	<0.001
Emotional Clarity	Total effect (c)	−0.223	0.059	−0.338 to −0.107	<0.001
Emotional Clarity	Direct effect (c′)	−0.035	0.057	−0.147 to 0.078	0.547
Emotional Clarity	Indirect effect (a × b)	−0.188	0.034	−0.259 to −0.125	—
Emotional Repair	Path a: Repair → Self-esteem	0.185	0.025	0.136 to 0.234	<0.001
Emotional Repair	Path b: Self-esteem → Bullying	−1.040	0.105	−1.246 to −0.834	<0.001
Emotional Repair	Total effect (c)	−0.140	0.060	−0.258 to −0.022	0.020
Emotional Repair	Direct effect (c′)	0.052	0.058	−0.061 to 0.165	0.363
Emotional Repair	Indirect effect (a × b)	−0.193	0.037	−0.270 to −0.125	—

Note: Sex was included as a covariate (girls = 1). Indirect effects were estimated using 5000 bootstrap resamples and were considered significant when the 95% confidence interval did not include zero. Regression and mediation results are based on 435 complete matched records available in the supplied datasets.

## Data Availability

The data presented in this study are available upon request to the corresponding author. As the dataset contains sensitive information collected from minors regarding school bullying, self-esteem, and emotional intelligence, the data cannot be made publicly available in order to protect participants’ privacy and comply with ethical and data protection requirements. However, anonymized data may be made available by the corresponding author upon reasonable request, subject to approval by the relevant Ethics Committee and compliance with applicable data protection regulations.
